# Population-Level Survival Trends in Hematologic Malignancies: A Surveillance, Epidemiology, and End Results (SEER) Analysis of 494,244 Patients From 2000 to 2020

**DOI:** 10.7759/cureus.111845

**Published:** 2026-06-30

**Authors:** Kevin Woodson, Julio Alvarenga

**Affiliations:** 1 Internal Medicine, Independent Research, Norman, USA; 2 Hematology and Oncology, City of Hope, Duarte, USA

**Keywords:** health disparities, hematologic malignancies, leukemia, lymphoma, multiple myeloma, population-based study, seer, survival outcomes

## Abstract

Population-level survival gains from transformative advances in hematologic malignancy treatment may not be equitably distributed. We examined over two decades of survival trends for acute myeloid leukemia (AML), acute lymphoblastic leukemia (ALL), non-Hodgkin lymphoma (NHL), and multiple myeloma using data from the National Cancer Institute (NCI) Surveillance, Epidemiology, and End Results (SEER) 17 Registries (2000-2020). Kaplan-Meier analysis estimated overall survival (OS) for 423,982 patients aged ≥15 years, stratified by diagnosis year, race/ethnicity, age, sex, and stage; observed OS reflects all-cause mortality. Multivariate Cox proportional hazards regression was performed on 494,244 patients to identify independent correlates of all-cause mortality. Five-year OS improved substantially from 2000 to 2017: myeloma (29.0%→53.7%, +85% relative improvement), ALL (30.7%→53.0%, +73%), AML (15.2%→27.9%, +84%), and NHL (54.5%→65.6%, +20%). Age ≥70 years was the strongest correlate of mortality (hazard ratio (HR): 7.20; 95% CI: 7.01-7.39 vs. <40 years). Non-Hispanic Black and Hispanic patients had significantly higher mortality than non-Hispanic White patients (HR: 1.22 and 1.11, respectively; both: p < 0.001). Each successive diagnosis period was independently associated with lower mortality (HR: 0.72 for 2015-2020 vs. 2000-2004). Survival has improved substantially but unequally across all four malignancies. Age and race/ethnicity remain independent correlates of mortality after full adjustment, warranting targeted interventions.

## Introduction

Hematologic malignancies, including acute myeloid leukemia (AML), acute lymphoblastic leukemia (ALL), non-Hodgkin lymphoma (NHL), and multiple myeloma, collectively represent one of the most significant burdens in oncology, accounting for a substantial proportion of cancer incidence worldwide [1]. In 2026, an estimated 22,720 new cases of AML, 6,250 new cases of ALL, 36,000 new cases of multiple myeloma, and approximately 79,320 new cases of NHL are projected in the United States alone, collectively accounting for approximately 6.8% of all new cancer diagnoses (144,290 of 2,114,850) [2]. Despite advances in therapy, these malignancies remain associated with substantial mortality, and the gap between outcomes in controlled clinical trial settings and real-world population-level practice continues to be an area of active investigation.

The therapeutic landscape for hematologic malignancies has been transformed over the past two decades by a series of landmark approvals. For multiple myeloma, proteasome inhibitors (bortezomib, 2003) and immunomodulatory agents (lenalidomide, 2006) redefined the standard of care [3]. Rituximab-based chemoimmunotherapy became first-line therapy for most NHL subtypes in the early 2000s [4], and Bruton's tyrosine kinase (BTK) inhibitors such as ibrutinib have extended treatment options for relapsed or refractory NHL subtypes [5]. For AML, FMS-like tyrosine kinase 3 (FLT3) inhibitors (midostaurin, 2017), isocitrate dehydrogenase (IDH) inhibitors, and venetoclax-based combinations have expanded treatment options, particularly for older or unfit patients [6]. For ALL, the introduction of tyrosine kinase inhibitors dramatically improved outcomes in Philadelphia chromosome-positive disease [7]. Whether these therapeutic advances have translated to meaningful and equitable population-level gains requires population-based evidence.

Most published survival data for hematologic malignancies are derived from single-institution case series, cooperative group trials, or disease-specific registries. While invaluable for characterizing outcomes in selected patient populations, these sources are subject to selection bias and may underrepresent older patients, racial and ethnic minorities, and individuals in geographically underserved areas [8]. Population-based data from the National Cancer Institute (NCI) Surveillance, Epidemiology, and End Results (SEER) Program [9,10] offer a complementary perspective, capturing outcomes across the full spectrum of patients with these diagnoses regardless of treatment received, and thus reflecting the real-world impact of therapeutic innovation at the societal level.

Despite the wealth of data available through SEER, longitudinal population-level analyses examining survival trends across multiple hematologic malignancies simultaneously, with attention to demographic disparities, remain limited. Prior studies have tended to focus on single disease types, shorter time periods, or specific demographic subgroups [11-13], leaving a gap in our understanding of how survival has evolved across two decades of therapeutic innovation and whether improvements have been equitably distributed.

In this study, we used data from the SEER 17 Registries database (2000-2020) to examine trends in overall survival (OS) for patients diagnosed with AML, ALL, NHL, or multiple myeloma. The primary objective was to characterize population-level temporal trends in OS across two decades of therapeutic innovation. Secondary objectives were to describe survival disparities by race/ethnicity, age group, sex, and stage at diagnosis, and to identify independent demographic and disease-level correlates of all-cause mortality using multivariable Cox proportional hazards regression. Our findings provide a comprehensive population-level picture of over two decades of progress and persistent inequity in the management of hematologic malignancies in the United States.

## Materials and methods

Study design

We conducted a retrospective, population-based cohort study using data from the NCI SEER Program. The study period spanned January 1, 2000, through December 31, 2020. This study utilized publicly available, de-identified data and did not require institutional review board approval.

Data source

Data were obtained from the Incidence-SEER Research Data, 17 Registries, Nov 2024 Sub (2000-2022) [9], which covers approximately 26.5% of the United States population across 17 geographic regions. The SEER program has collected data on cancer incidence, patient demographics, tumor characteristics, and survival since 1973 and is widely recognized as the gold standard for population-level cancer epidemiology research in the United States [9]. Data were accessed and analyzed using SEER*Stat software (version 9.0.43.0) (released date: 2026; National Cancer Institute, Surveillance Systems Branch; Bethesda, MD, USA) [14], the NCI’s dedicated statistical tool for SEER database analysis. All analyses were performed in accordance with the SEER data use agreements.

Study population

Eligible patients included patients aged 15 years or older diagnosed with one of four hematologic malignancies between January 1, 2000, and December 31, 2020. Cancer types were identified using the Site Recode International Classification of Diseases for Oncology, 3rd Edition (ICD-O-3)/World Health Organization (WHO) 2008 classification system [9]. The four cancer types included were AML, ALL, NHL, and multiple myeloma.

Cases with unknown age, diagnoses recorded via death certificate only, or alive with no recorded survival time were excluded using SEER’s standard case selection criteria. The lower age boundary of 15 years reflects SEER’s five-year age grouping structure; the 15-19-year group includes a small proportion of adolescents aged 15-17, which is noted as a limitation. Analyses were restricted to patients with malignant behavior and known age at diagnosis.

Variables

Primary Outcome

The primary outcome was observed OS, defined as the time from diagnosis to death from any cause or last follow-up, whichever occurred first. Survival was estimated at 12 months (one year), 36 months (three years), and 60 months (five years) time points using the Kaplan-Meier method.

Exposure Variables

The following patient- and tumor-level variables were examined as stratification factors: year of diagnosis (2000-2020, analyzed annually as a proxy for treatment era), race/ethnicity (non-Hispanic White, non-Hispanic Black, non-Hispanic American Indian/Alaska Native, non-Hispanic Asian or Pacific Islander, and Hispanic, using SEER’s recommended race and origin recode variable), age group at diagnosis (15-39, 40-59, 60-74, and 75+ years, derived from SEER’s five-year age recode), sex (male, female), and stage at diagnosis (localized, regional, distant, Unknown/Unstaged, using SEER Summary Stage 2000, covering cases diagnosed through 2017; AML and ALL are not assigned localized/regional/distant staging under this system; therefore, leukemia cases without applicable stage were included in the Unknown/Unstaged category for the pooled Cox model, and stage-related hazard ratios (HRs) should be interpreted cautiously as they primarily reflect staged NHL and myeloma cases)

Statistical analysis

OS was estimated using the Kaplan-Meier method with 95% confidence intervals (CIs) calculated for all survival estimates. Observed survival (all-cause mortality) was used as the primary survival measure, rather than relative or cause-specific survival, to reflect the clinical reality of overall treatment burden in this population.

Survival estimates were stratified by each exposure variable independently, with cancer type (site recode) included as the primary row variable in all analyses. This approach produced five separate stratified analyses: (1) cancer type by year of diagnosis, (2) cancer type by race/ethnicity, (3) cancer type by age group, (4) cancer type by sex, and (5) cancer type by stage at diagnosis.

For the age analysis, SEER’s five-year age recode groups were collapsed into four clinically meaningful categories (15-39, 40-59, 60-74, 75+) by aggregating adjacent age bands into each category. Cox regression used a slightly different grouping (<40, 40-59, 60-69, ≥70 years) to align with clinically meaningful mortality thresholds; both schemes are described where applicable. All analyses were performed using SEER*Stat software. Descriptive statistics and data visualization were performed using Python (pandas, matplotlib). No imputation was performed for missing data; cases with missing stage information are reported in the "Unknown/Unstaged" category.

To identify independent correlates of all-cause mortality, a multivariate Cox proportional hazards regression was performed using a patient-level case listing exported from SEER*Stat (N = 494,244; 292,553 events; median follow-up 49 months). Covariates entered simultaneously into the model were cancer type (AML as reference), age group (<40 as reference), sex (female as reference), race/ethnicity (non-Hispanic White as reference), calendar period of diagnosis (2000-2004 as reference), and stage at diagnosis (localized as reference). A ridge penalty (λ = 0.005) was applied to address near-collinearity among categorical covariates and to ensure numerical stability, particularly for small racial/ethnic subgroups. HRs and 95% CIs were derived from the Breslow partial likelihood with Newton-Raphson optimization. Statistical significance was set at p < 0.05 (two-tailed). Formal testing of the proportional hazards assumption was not performed; HRs are interpreted as time-averaged estimates. Because AML and ALL are not assigned localized/regional/distant Summary Stage in SEER, leukemia cases without applicable stage were included in the Unknown/Unstaged category in the pooled Cox model; therefore, stage-related HRs should be interpreted cautiously and primarily reflect staged NHL and myeloma cases.

## Results

Study population

A total of 423,982 patients aged 15 years or older were identified in the SEER 17 Registries survival session with a diagnosis of AML, ALL, NHL, or multiple myeloma between 2000 and 2020, used for Kaplan-Meier analyses. A separate case listing export of 494,244 patients was used for multivariate Cox regression; the difference reflects internal record-selection differences between SEER*Stat session types. NHL accounted for the largest proportion of cases (N = 271,268; 64.0%), followed by myeloma (N = 94,309; 22.2%), AML (N = 45,732; 10.8%), and ALL (N = 12,673; 3.0%). Non-Hispanic White patients comprised the majority of the cohort (67.4%); male patients accounted for 54.4%.

Survival trends over time (2000-2017)

**Figure 1:**
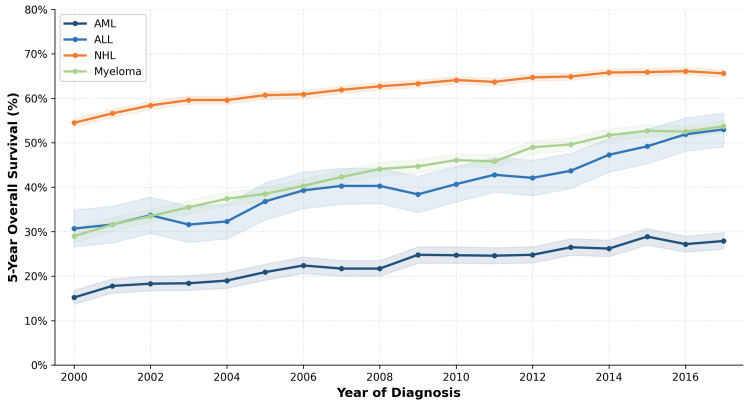
Trends in five-year overall survival by hematologic malignancy, SEER 17 Registries (2000-2017) SEER: Surveillance, Epidemiology, and End Results Shaded bands represent 95% confidence intervals

Five-year OS improved substantially across all four hematologic malignancies between 2000 and 2017 (the most recent year with complete five-year follow-up data), as shown in Figure 1. The most dramatic absolute improvement was observed for multiple myeloma (five-year OS: 29.0% in 2000 → 53.7% in 2017; +24.7 percentage points (pp); 85% relative improvement), followed by ALL (30.7% → 53.0%; +22.3 pp; 73%), AML (15.2% → 27.9%; +12.7 pp; 84%), and NHL (54.5% → 65.6%; +11.1 pp; 20%) (Table 1). Despite these gains, AML remained associated with the lowest absolute five-year OS of any cancer type throughout the study period.

**Table 1:** Overall survival at 1, 3, and 5 years by cancer type: 2000 vs. 2017 OS: overall survival; pp: percentage points

Cancer type	1-year OS2000	3-year OS2000	5-year OS2000	1-year OS2017	3-year OS2017	5-year OS2017	Change (5 years)
Acute myeloid leukemia (AML)	33.3%	18.2%	15.2%	49.4%	32.3%	27.9%	+12.7pp/+84%
Acute lymphoblastic leukemia (ALL)	56.2%	37.7%	30.7%	76.7%	58.4%	53.0%	+22.3pp/+73%
Non-Hodgkin lymphoma (NHL)	75.5%	62.3%	54.5%	81.3%	72.2%	65.6%	+11.1pp/+20%
Myeloma	69.9%	44.5%	29.0%	81.9%	67.1%	53.7%	+24.7pp/+85%

Racial and ethnic disparities

**Figure 2:**
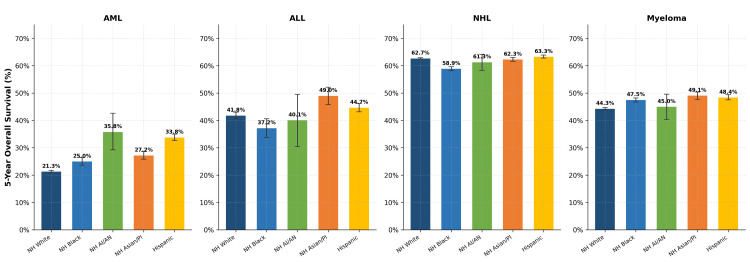
Five-year overall survival by race/ethnicity and hematologic malignancy, SEER 17 Registries (2000-2020) SEER: Surveillance, Epidemiology, and End Results; NH: non-Hispanic; AI/AN: American Indian/Alaska Native; PI: Pacific Islander Error bars represent 95% confidence intervals

Racial and ethnic disparities in five-year OS were present across all cancer types, though the magnitude and direction of disparities varied by diagnosis (Figure 2). For AML, non-Hispanic White patients had the lowest five-year OS (21.3%), a notable and counterintuitive finding, compared with non-Hispanic American Indian/Alaska Native patients (35.8%) and Hispanic patients (33.8%). The racial gap in AML five-year OS was 14.5 pp between the highest- and lowest-performing groups.

For ALL, non-Hispanic Black patients had the lowest five-year OS (37.2%), compared with non-Hispanic Asian/Pacific Islander patients (49.0%), representing an 11.8 pp disparity. For NHL, racial disparities were relatively modest, with a 4.4 pp gap between Hispanic patients (63.3%) and non-Hispanic Black patients (58.9%). For myeloma, non-Hispanic Asian/Pacific Islander patients had the highest five-year OS (49.1%), while non-Hispanic White patients had the lowest (44.3%), a 4.8 pp gap.

Age-based differences

**Figure 3:**
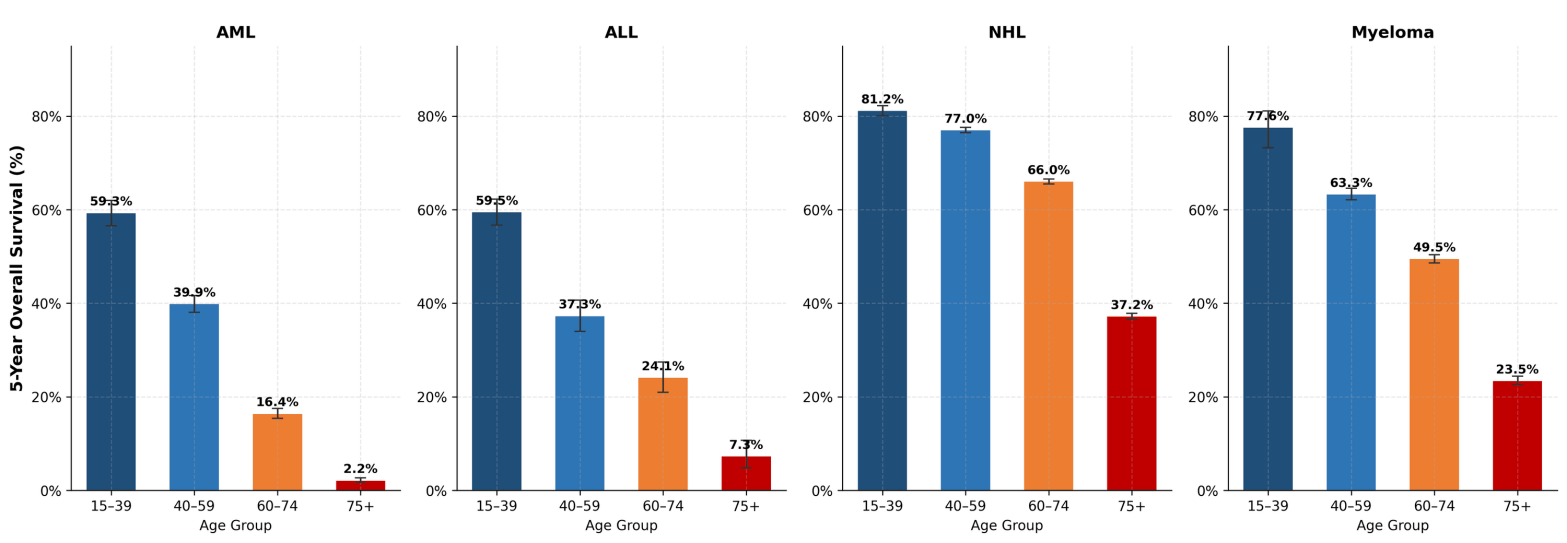
Five-year overall survival by age group and hematologic malignancy, SEER 17 Registries (2000-2020) Error bars represent 95% confidence intervals

Age at diagnosis was the strongest correlate of survival across all four cancer types (Figure 3). A steep and consistent age gradient was observed, with younger patients achieving substantially higher survival rates than older patients. For AML, the five-year OS ranged from 59.3% in patients aged 15-39 years to just 2.2% in patients aged 75 or older, a 57.1 pp gap. Similarly large disparities were observed for ALL (59.5% vs. 7.3%; 52.2 pp gap), myeloma (77.6% vs. 23.5%; 54.1 pp gap), and NHL (81.2% vs. 37.2%; 44.0 pp gap). These findings highlight the profound impact of age on treatment tolerance and outcomes in hematologic malignancies.

Sex differences

**Figure 4:**
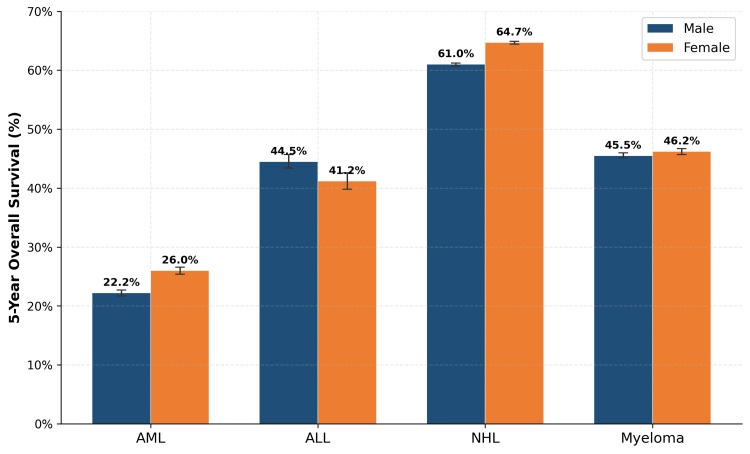
Five-year overall survival by sex and hematologic malignancy, SEER 17 Registries (2000-2020) Error bars represent 95% confidence intervals

Sex-based differences in five-year OS were modest but consistent across cancer types (Figure 4). Female patients had superior survival compared with male patients for AML (26.0% vs. 22.2%; +3.8 pp), NHL (64.7% vs. 61.0%; +3.7 pp), and myeloma (46.2% vs. 45.5%; +0.7 pp). The exception was ALL, in which male patients had marginally higher five-year OS (44.5% vs. 41.2%; +3.3 pp favoring males).

Stage at diagnosis

**Figure 5:**
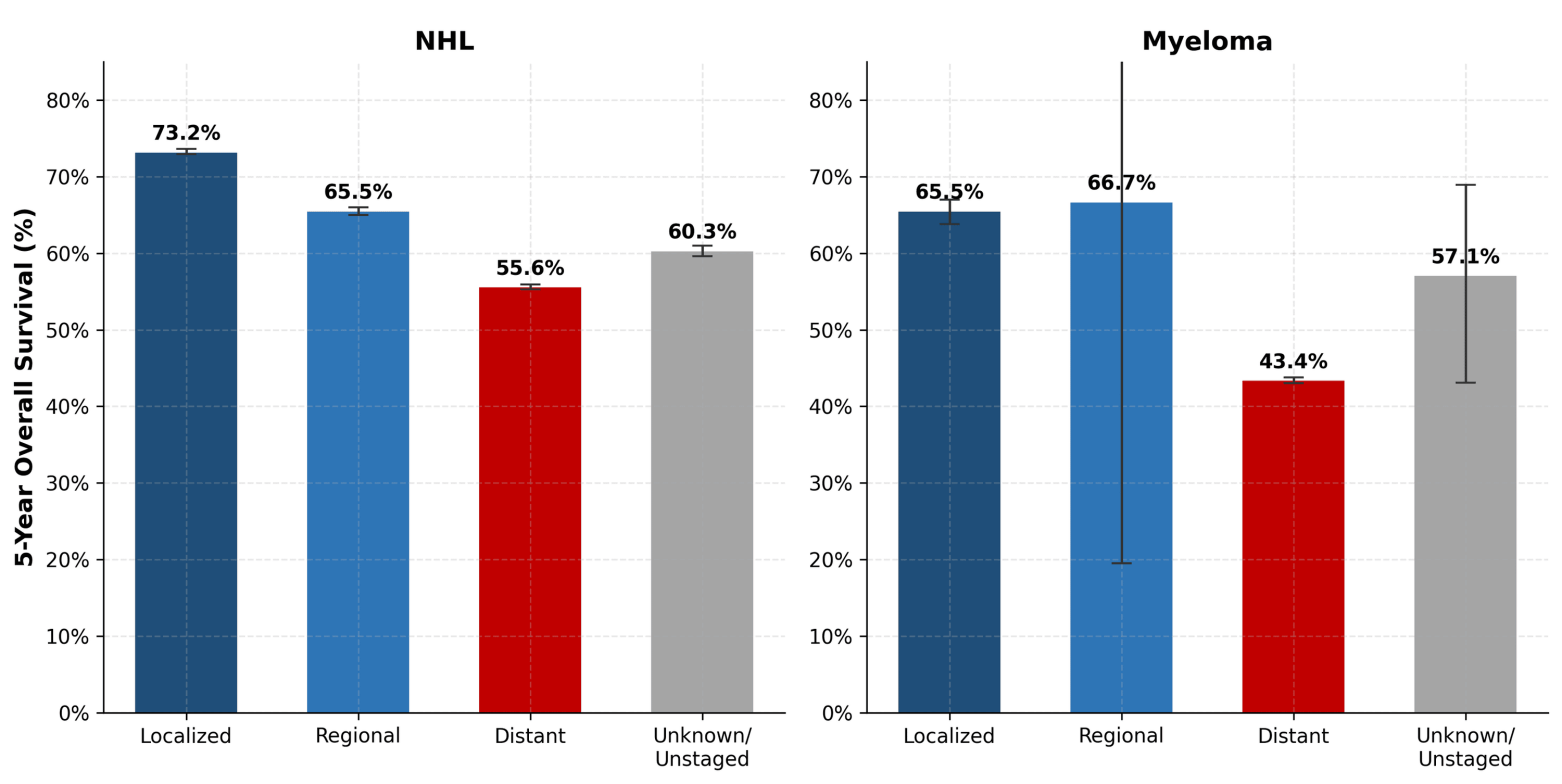
Five-year overall survival by stage at diagnosis, NHL and myeloma, SEER summary stage 2000 (2000-2017) Error bars represent 95% confidence intervals. Hatched bars indicate categories with small sample sizes; estimates should be interpreted with caution. AML and ALL are not assigned localized/regional/distant staging in SEER

Stage at diagnosis was analyzed for NHL and myeloma using SEER Summary Stage 2000 (applicable to cases diagnosed through 2017). AML and ALL are not classified using the localized/regional/distant staging system in SEER. For NHL, five-year OS decreased with advancing stage: localized (73.2%), regional (65.5%), and distant (55.6%), a 17.6 pp gap between localized and distant disease (Figure 5). For myeloma, localized disease had a five-year OS of 65.5%, while distant disease had substantially lower survival (43.4%), a 22.1 pp gap. The regional-stage myeloma category included only six patients and is not considered interpretable.

Multivariate Cox proportional hazards analysis

**Figure 6:**
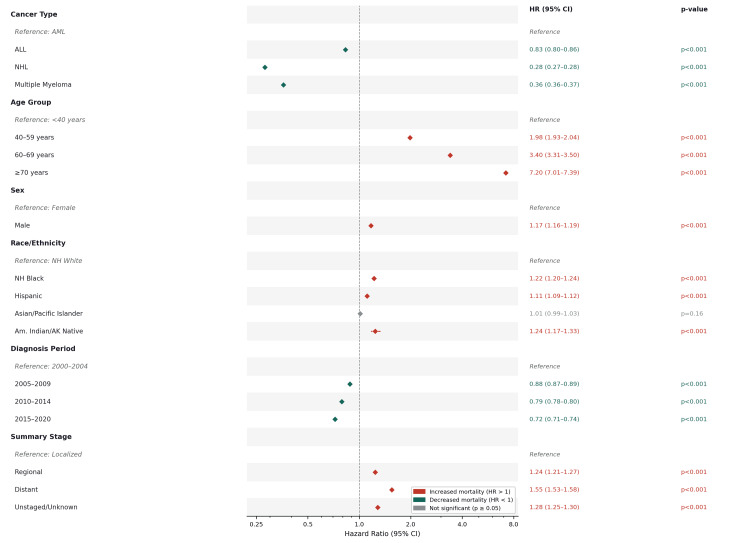
Multivariate Cox proportional hazard regression: correlates of all-cause mortality in hematologic malignancies (SEER 2000-2020) AML: acute myeloid leukemia; SEER: Surveillance, Epidemiology, and End Results N = 494,244 patients; 292,553 events. Reference categories: AML, age <40 years, female sex, non-Hispanic White, diagnosis period 2000-2004, localized stage. All hazard ratios adjusted simultaneously for all covariates

A multivariate Cox proportional hazards regression was performed on 494,244 patients (292,553 events; 59.2% mortality; median follow-up 49 months), adjusting simultaneously for cancer type, age group, sex, race/ethnicity, diagnosis period, and stage. The model converged in six iterations. Figure 6 presents the forest plot of HRs.

Cancer type was a highly significant correlate after adjustment for all other covariates. Compared with AML (reference), NHL demonstrated a 72% lower mortality risk (HR: 0.28; 95% CI: 0.27-0.28), myeloma a 64% lower risk (HR: 0.36; 95% CI: 0.36-0.37), and ALL a 17% lower risk (HR: 0.83; 95% CI: 0.80-0.86), all p < 0.001.

Age at diagnosis was the strongest independent correlate of mortality in the model. Compared with patients younger than 40 years, those aged 40-59 had nearly twice the mortality risk (HR: 1.98; 95% CI: 1.93-2.04), those aged 60-69 had more than three times the risk (HR: 3.40; 95% CI: 3.31-3.50), and those aged 70 or older had more than seven times the risk (HR: 7.20; 95% CI: 7.01-7.39), all p < 0.001. This age gradient persisted after adjustment for all other covariates, confirming that age is an independent predictor of mortality. It should be noted that all-cause mortality was used as the primary endpoint; in older patients, particularly those aged ≥70 years, competing causes of death unrelated to hematologic malignancy may contribute to the observed mortality differences, and cancer-specific survival estimates may differ from those reported here. Male sex was independently associated with a 17% higher mortality risk compared with female patients (HR: 1.17; 95% CI: 1.16-1.19; p < 0.001).

Race/ethnicity remained an independent correlate of mortality after adjustment for age, stage, cancer type, and diagnosis period. Compared with non-Hispanic White patients (reference), non-Hispanic Black patients had a 22% higher mortality risk (HR 1.22; 95% CI: 1.20-1.24; p < 0.001), and Hispanic patients had an 11% higher risk (HR: 1.11; 95% CI: 1.09-1.12; p < 0.001). Non-Hispanic American Indian/Alaska Native patients also had significantly higher mortality (HR: 1.24; 95% CI: 1.17-1.33; p < 0.001), though this group was small (n = 2,875) and estimates should be interpreted with caution. Non-Hispanic Asian/Pacific Islander patients had mortality risk statistically indistinguishable from non-Hispanic White patients (HR: 1.01; 95% CI: 0.99-1.03; p = 0.16). These findings indicate that racial disparities in survival are not fully explained by differences in age, cancer type, stage, or era of diagnosis.

Diagnosis period demonstrated a consistent and progressive reduction in mortality risk over calendar time after adjustment for all other covariates. Compared with patients diagnosed in 2000-2004, those diagnosed in 2005-2009 had a 12% lower mortality risk (HR: 0.88; 95% CI: 0.87-0.89), those diagnosed in 2010-2014 a 21% lower risk (HR: 0.79; 95% CI: 0.78-0.80), and those diagnosed in 2015-2020 a 28% lower risk (HR: 0.72; 95% CI: 0.71-0.74), all p < 0.001. This finding is consistent with population-level gains coinciding with the modern treatment era, persisting after adjustment for measured demographic and disease characteristics.

Stage at diagnosis was independently associated with mortality. Compared with localized disease, regional disease (HR: 1.24; 95% CI: 1.21-1.27), distant disease (HR: 1.55; 95% CI: 1.53-1.58), and Unstaged/Unknown cases (HR: 1.28; 95% CI: 1.25-1.30) all carried significantly higher mortality risk (all p < 0.001).

## Discussion

This population-based analysis of 494,244 patients demonstrates substantial improvements in overall survival across all four hematologic malignancies over two decades, alongside persistent disparities by age, race/ethnicity, and sex. These findings identify the subgroups that have benefited least from therapeutic innovation at the population level.

Survival improvements and the role of novel therapeutics

The most dramatic survival gains observed in this analysis were for multiple myeloma (+85% relative improvement in five-year OS from 2000 to 2017) and ALL (+73%). These improvements coincide with the introduction and widespread adoption of transformative therapies during this period. For myeloma, the approval of bortezomib (2003) and lenalidomide (2006) marked a paradigm shift from conventional chemotherapy to proteasome inhibitor- and immunomodulatory drug-based regimens. Population-level analyses using SEER-Medicare data have confirmed that adoption of these novel agents was directly associated with improved OS at the population level [15,16].

For NHL, improvements in five-year OS (+20%) are consistent with the widespread adoption of rituximab-based chemoimmunotherapy, particularly rituximab, cyclophosphamide, hydroxydaunorubicin, oncovin, and prednisone (R-CHOP), which became the standard of care for diffuse large B-cell lymphoma (DLBCL) following landmark trials published in the early 2000s [4]. Population-based analyses have confirmed that rituximab favorably altered the long-term prognosis of NHL patients at the population level [4,12]. The relatively smaller relative improvement for NHL compared with myeloma and ALL likely reflects the fact that NHL already had a more favorable baseline survival (54.5% in 2000), leaving less room for improvement. The persistently low absolute survival for AML underscores the ongoing unmet need in this disease [17-19].

Racial and ethnic disparities

Notably, non-Hispanic White patients with AML had the lowest five-year OS (21.3%) of any racial/ethnic group, a counterintuitive finding that likely reflects unmeasured biological factors not captured in SEER, including cytogenetic risk category and mutational profile. The relationship between race and AML outcome may be further modified by differential access to high-volume treatment centers and clinical trials [18,19].

For ALL, the 11.8 pp survival gap between non-Hispanic Asian/Pacific Islander patients (49.0%) and non-Hispanic Black patients (37.2%) is consistent with broader literature documenting race-, age-, and trial-participation disparities in hematologic malignancies [8,20]. Potential contributors include differential access to specialized centers, treatment intensity, insurance-related barriers, and underrepresentation in clinical trials, although these mechanisms cannot be directly assessed in SEER.

Notably, NHL showed the smallest racial/ethnic disparities of any cancer in this analysis (4.4 pp gap), suggesting that the widespread adoption of rituximab-based chemoimmunotherapy may have had a relatively equalizing effect across racial groups. This hypothesis is consistent with prior work demonstrating broad outcome improvements with rituximab-based therapy in DLBCL and NHL populations [4,21]. Continued monitoring of disparities as chimeric antigen receptor T-cell (CAR-T cell) therapy and bispecific antibodies become standard of care for relapsed NHL will be important.

For myeloma, the racial disparities observed in this analysis, with non-Hispanic White patients having the lowest five-year OS (44.3%) and non-Hispanic Asian/Pacific Islander patients the highest (49.1%), are consistent with prior population-based literature. Waxman et al. demonstrated that while Black patients have more than twice the incidence of myeloma compared with White patients, disease-specific and relative survival rates were paradoxically higher in Black patients over the study period, a pattern that has persisted into the novel agent era and may reflect biological differences in tumor characteristics or differential benefit from proteasome inhibitor-based regimens [22].

Age as the dominant correlate of survival

Age at diagnosis was the single strongest correlate of survival across all four cancer types in this analysis, with a 57-percentage-point gap in five-year OS for AML between patients aged 15-39 years (59.3%) and those aged 75 or older (2.2%). This dramatic gradient reflects the profound impact of age on multiple determinants of outcome: fitness for intensive induction chemotherapy, tolerance of treatment-related toxicity, the higher prevalence of adverse cytogenetics in older patients, and the greater burden of comorbidities.

Population-based data from SEER confirm five-year relative survival of approximately 46%, 26%, 11%, and 3% for AML patients aged 20-49, 50-64, 65-74, and 75 or older, respectively, closely mirroring our findings [17,23]. The sharp mortality gradient with age likely reflects multiple compounding factors: lower fitness for intensive induction chemotherapy, higher rates of adverse-risk cytogenetics in older patients, greater comorbidity burden, and differential access to specialized oncology centers.

These findings underscore the urgent need for age-adapted treatment strategies and greater integration of geriatric oncology principles into the management of hematologic malignancies. The development of less toxic, highly effective regimens, such as venetoclax-based combinations for AML and oral targeted therapies for myeloma, holds genuine promise for improving outcomes in older patients who are currently underserved by intensive treatment paradigms, and the progressive reduction in mortality hazard by diagnosis period observed in this analysis suggests these efforts are beginning to bear fruit at the population level.

Sex-based differences

Sex-based differences in five-year OS were modest but consistent: female patients had superior survival for AML (+3.8 pp), NHL (+3.7 pp), and myeloma (+0.7 pp), while male patients had marginally better five-year OS for ALL (+3.3 pp). These differences may reflect disease-specific biology, treatment tolerance, comorbidity patterns, and background mortality differences, although SEER does not allow these mechanisms to be directly evaluated [13,18].

Stage at diagnosis

Stage-stratified analyses for NHL and myeloma demonstrated the expected inverse relationship between disease extent and survival. For NHL, the 17.6 pp gap in five-year OS between localized (73.2%) and distant disease (55.6%) highlights the prognostic importance of early detection. For myeloma, localized disease had a five-year OS of 65.5%, while distant disease had substantially worse outcomes (43.4%), a 22.1 pp gap. The regional-stage myeloma category included only six patients and was not considered interpretable. These patterns reflect the biologic heterogeneity of myeloma staging, in which the localized/regional distinction is less clinically meaningful than in solid tumors.

Limitations

This study has several important limitations that should be considered when interpreting these findings. First, SEER does not capture treatment data, including the specific agents administered, treatment intensity, or use of hematopoietic stem cell transplantation. Survival estimates therefore reflect the entire diagnosed population, and temporal trends cannot be attributed to any specific therapeutic intervention. Additionally, observed overall survival reflects all-cause mortality and therefore incorporates background population mortality; this is particularly relevant for older patient subgroups where competing causes of death are common, and survival estimates should not be equated with cancer-specific mortality.

Second, SEER lacks socioeconomic status, insurance status, and comorbidity data, which likely confound the racial and age-based disparities observed. Third, stage data using Summary Stage 2000 is available only through 2017, limiting stage-stratified analyses to pre-2018 cases. Fourth, the 15-19 year age group may include a small number of adolescent cases due to SEER's five-year grouping structure; more broadly, the 15-39 age group encompasses patients with considerable biological and clinical heterogeneity, as tumor biology, treatment protocols, and outcomes differ meaningfully across this range. Fifth, the American Indian/Alaska Native subgroup was small (n = 2,875), and Cox estimates should be interpreted with caution. Sixth, unmeasured confounding cannot be excluded. Seventh, localized/regional/distant Summary Stage is not applicable to AML or ALL; these cases were included in Unknown/Unstaged, and stage-related HRs primarily reflect staged NHL and myeloma cases. Eighth, NHL was analyzed as a single category; given the marked heterogeneity in prognosis across NHL subtypes (e.g., DLBCL, follicular lymphoma, mantle cell lymphoma), pooling across subtypes likely masks subtype-specific trends, and findings for NHL should be interpreted with this limitation in mind. Ninth, formal testing of the proportional hazards (PH) assumption was not performed; the pooled Cox model estimates time-averaged HRs across a 20-year follow-up period during which treatment paradigms changed substantially, and HRs may not remain constant over time for all covariates. Tenth, SEER does not capture hospital type, volume, or quality metrics; differential access to high-volume academic centers and specialized oncology expertise may contribute to the survival disparities observed and represents an unmeasured source of confounding. Eleventh, SEER does not include molecular or cytogenetic data, which are major determinants of prognosis and treatment selection, particularly for AML, where cytogenetic risk stratification (favorable, intermediate, and adverse) significantly influences survival outcomes independent of age and treatment era. Twelfth, geographic variation at the patient level, including urban versus rural residence and regional differences in access to care, is not captured in this analysis and may confound the observed disparities. Finally, the pooled Cox model does not capture disease-level heterogeneity; HRs for covariates such as age and race represent averages across AML, ALL, NHL, and myeloma, and the direction and magnitude of associations may differ substantially by cancer type.

Future directions

Future research should include joinpoint regression analyses to identify inflection points in survival trends corresponding to specific therapeutic approvals; SEER-Medicare linkage studies to examine socioeconomic factors, comorbidities, treatment receipt, and care access as mediators of racial and age-based disparities; and formal mediation analyses to estimate the proportion of racial survival gaps attributable to treatment access, healthcare delivery, and disease biology. As CAR-T cell therapy, bispecific antibodies, and next-generation targeted agents continue to transform the treatment landscape, ongoing population-level surveillance will be essential to ensure that these advances benefit patients equitably.

## Conclusions

This population-based analysis shows that survival for patients with hematologic malignancies has improved substantially over the past two decades, coinciding with the introduction and diffusion of novel therapeutic agents. However, these gains have not been distributed equally. Multivariable Cox regression confirmed that age was the dominant independent predictor of mortality, while progressively lower mortality hazards by calendar period were consistent with population-level gains across the modern treatment era. After adjustment for age, malignancy type, stage, and era of diagnosis, non-Hispanic Black and Hispanic patients continued to experience higher mortality risk than non-Hispanic White patients. These findings suggest that racial survival disparities are not fully explained by recorded demographic and disease factors alone and highlight the need for targeted interventions to improve equitable access to advances in hematologic cancer care.
